# Brillouin microspectroscopy data of tissue-mimicking gelatin hydrogels

**DOI:** 10.1016/j.dib.2020.105267

**Published:** 2020-02-08

**Authors:** Michelle Bailey, Noemi Correa, Simon Harding, Nick Stone, Sophie Brasselet, Francesca Palombo

**Affiliations:** a School of Physics and Astronomy, University of Exeter, Exeter, EX4 4QL, UK; b Machine Intelligence Ltd, South Zeal, EX20 2JS, UK; c Institut Fresnel, CNRS, Aix Marseille University, Marseille, F-13013, France

**Keywords:** Brillouin scattering, Phonons, Biopolymers, Tissue phantoms, Collagen, Biomechanics

## Abstract

Brillouin spectroscopy, based on the inelastic scattering of light from thermally driven acoustic waves or *phonons* [1], holds great promise in the field of life sciences as it provides functionally relevant micromechanical information in a contactless all-optical manner [2]. Due to the complexity of biological systems such as cells and tissues, which present spatio-temporal heterogeneities, interpretation of Brillouin spectra can be difficult. The data presented here were collected from gelatin hydrogels, used as tissue-mimicking model systems for Brillouin microspectroscopy measurements conducted using a lab-built Brillouin microscope with a dual-stage VIPA spectrometer. By varying the solute concentration in the range 4–18% (w/w), the macroscopic mechanical properties of the hydrogels can be tuned and the corresponding evolution in the Brillouin-derived longitudinal elastic modulus measured. An increase in Brillouin frequency shift with increasing solute concentration was observed, which was found to correlate with an increase in acoustic wave velocity and longitudinal modulus. The gels used here provide a viable model system for benchmarking and standardisation, and the data will be useful for spectrometer development and validation.

**Table:** Specifications Table

Subject	Biophysics
Specific subject area	Brillouin scattering spectroscopy
Type of data	Graph Figures Images Table
How data were acquired	Brillouin Microscopy: Olympus iX73 inverted microscope coupled to a cw 532 nm Cobolt Samba laser, lab-built dual-stage Virtually Imaged Phase Array (VIPA) spectrometer (two VIPA etalons; Light Machinery) and Andor ZYLA-4.2P-USB3 sCMOS camera
Data format	Raw Analysed
Parameters for data collection	Gelatin hydrogels were analysed at room temperature (20 °C), approximately 24 h after preparation.
Description of data collection	Gelatin hydrogels at a concentration ranging between 4 and 18% w/w were placed onto a glass cover slip and analysed using a lab-built Brillouin microscope with a 60× (NA 1.20) water immersion objective.
Data source location	University of Exeter Exeter UK 50.7184° N, 3.5339° W
Data accessibility	Repository name: Open Research Exeter (ORE), University of Exeter, UK Data identification number: 10.24378/exe.2144 Direct URL to data: https://doi.org/10.24378/exe.2144

**Table:** 

**Value of the Data** • These data relate to gelatin hydrogels derived from denatured collagen that are biologically relevant homogeneous materials, useful to extract and understand the information contained within Brillouin spectra. • They can benefit the whole BioBrillouin community, providing a benchmark for testing and validation of instruments. They can also benefit life scientists, biologists and clinicians who are interested in novel biophotonic techniques. • In addition, these data can be used to draw comparisons between similar lab-built spectrometers, to gain further insights and to promote the development of new concepts for faster high-contrast, high-resolution Brillouin spectroscopy. • The use of transparent homogeneous materials that are reasonably stable at ambient conditions adds additional value to these data for system benchmarking and standardisation.

## 1 Data description

**Fig. 1:**
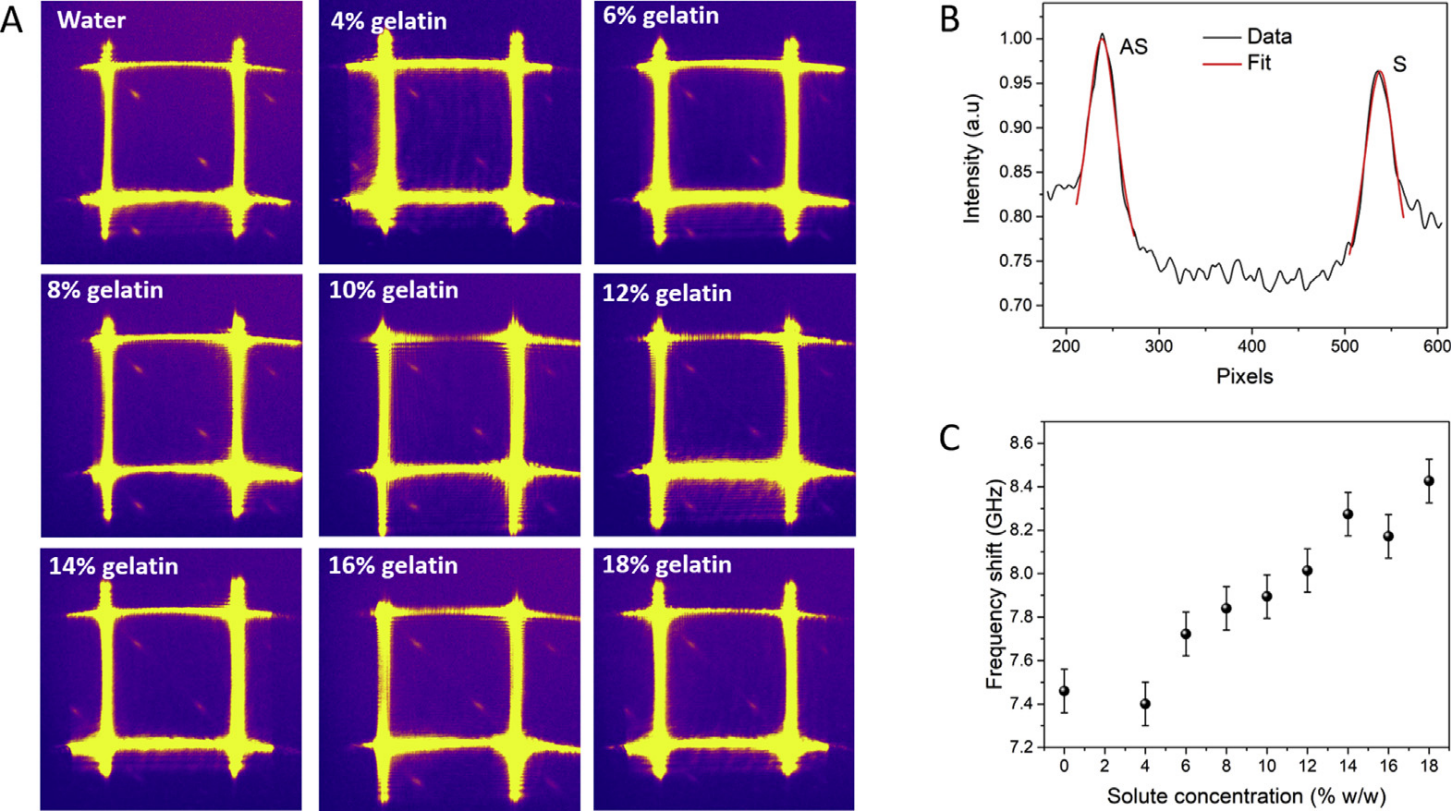
(A) Pseudo-colour images of the sCMOS outputs for gelatin hydrogels at varying solute concentration, from 0 to 18% w/w. (B) Spectrum of an 8% gelatin hydrogel before calibration (black line) and Lorentzian fit for both anti-Stokes (AS) and Stokes (S) peaks (red line; R^2^ = 0.97). (C) Plot of the Brillouin frequency shift vs. solute concentration of the gelatin hydrogels. Error bars account for drift in the calibration spectra during the course of the experiment and encompass intra-sample variability.

Microspectroscopic data of gelatin hydrogels at varying solute concentration up to 18% (w/w) were acquired using a lab-built Brillouin microscope with a two-stage VIPA spectrometer previously described [3] (see Table 1 for full specifications). Pseudo-colour images for different solute concentrations are presented in Fig. 1A. A Brillouin spectrum was extracted from each raw image and fit analysis to a Lorentzian function was applied to both Stokes and anti-Stokes peaks (Fig. 1B) arising from the interation of light with acoustic waves or *phonons* [1]. Average peak parameters were calculated (see Methods and Fig. 3) and Fig. 1C shows a plot of the Brillouin frequency shift as a function of solute concentration.

**Table 1:** VIPA-Brillouin microscope system specifications.

Parameter	Value
Laser wavelength	532 nm
Laser power (on the sample)	6 mW
Laser spectral linewidth (FWHM)	<1 MHz
Scattering geometry	180°
Objective lens	60x (NA 1.2) WI
Free spectral range	33 ± 2 GHz
Spectral resolution	0.9 ± 0.1 GHz
Finesse	38 ± 6
SNR	17 dB (methanol)

**Fig. 2:**
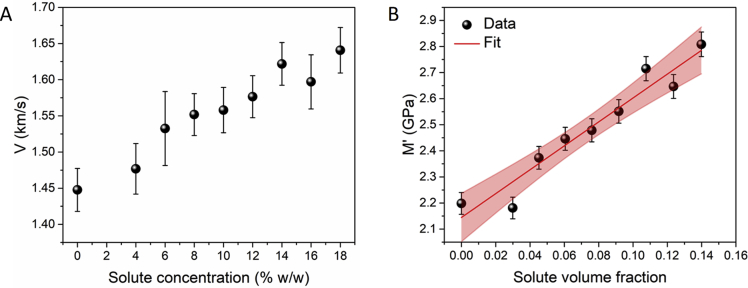
Plot of (A) acoustic wave velocity V vs. solute concentration and (B) longitudinal elastic modulus $M^{'}$ vs. solute volume fraction. Red line: fit to the Voigt model applied to the data, M'=6.73x+2.14(1−x); R^2^ = 0.92. Shading: 95% confidence band of the fit.

Using these data, the acoustic wave velocity V was determined (see below; Fig. 2A), which reproduces values previously found in the cornea and lens of the eye [4], and from this the longitudinal elastic modulus $M^{'}$ was derived. As already noted [2], the longitudinal modulus derived from Brillouin scattering includes the contribution of the adiabatic bulk modulus that is of the order of GPa even in water. A plot of $M^{'}$ vs. solute volume fraction x enables the Voigt model (rule of mixing) [5,6] to be applied to these data (Fig. 2B) according to the expression:

(1) $$M^{'}=M_{s}x+M_{w}\left(1-x\right)$$

where $M_{s}$ and $M_{w}$ are the longitudinal elastic moduli of the solute (gelatin) and water, respectively.

**Fig. 3:**
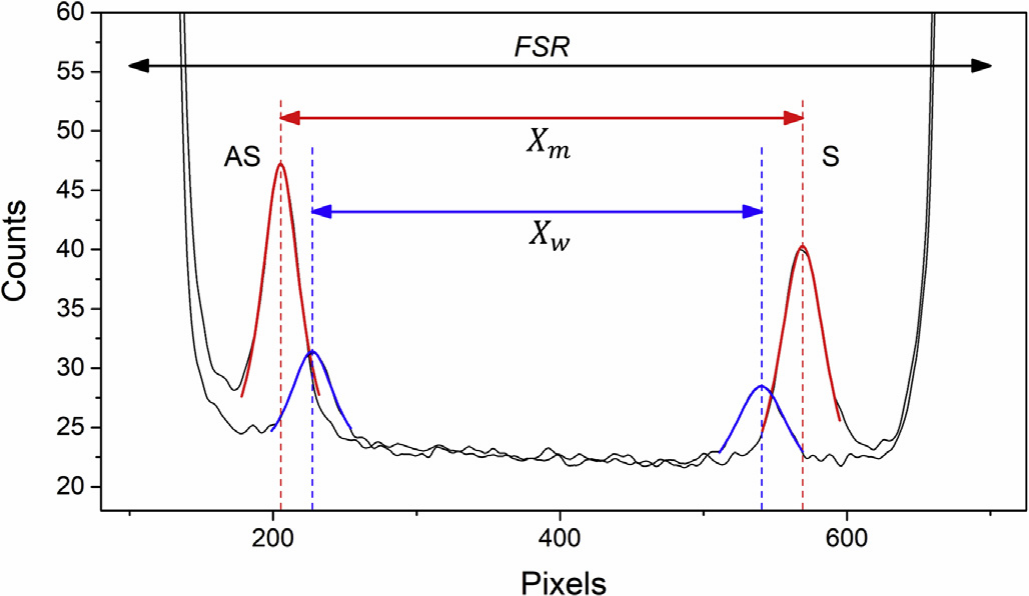
Calibration spectra and Lorentzian fit for methanol (red) and water (blue). Distances between Brillouin peaks, $X_{m}$ and $X_{w}$, were used to determine absolute peak positions. The free spectral range (FSR) between adjacent Rayleigh peaks is shown.

All the data presented in this article are available for download and reuse from the Open Research Exeter (ORE) repository, University of Exeter UK.

## 2 Experimental design, materials, and methods

### 2.1 Hydrogel preparation

Gelatin from bovine skin, gel strength ∼225 g Bloom, Type B (G9382, Sigma-Aldrich) was used to prepare hydrogels with solute concentration in the range 4–18% (w/w). Gelatin powder was combined with the appropriate quantity of distilled water to a total mass of 20 g and the mixture was held in a water bath (temperature 55–65 °C) for 60 min under magnetic stirring. This was sufficient time for the gelatin powder to fully dissolve at all concentrations prepared. All gels were left to set at room temperature, covered in parafilm to reduce evaporation and measured approximately 24 h after gelation, as preliminary testing established that this was sufficient time for the gels to stabilise. A small rectangular piece of gel (<10 mm thickness) was cut from the bulk and placed on a round glass coverslip (Biochrom, 0.17 mm thickness) in an Attofluor cell chamber (Life Technologies) for microscopy measurements.

### 2.2 Brillouin measurements

Brillouin microscopy measurements were conducted using a lab-built setup [3] developed on the basis of previous works [7,8], comprised of a 532 nm cw laser (Cobolt Samba), inverted microscope (Olympus iX73) with 60× (NA 1.20) water immersion objective (Olympus UPlanApo), and dual-stage VIPA (LightMachinery, 30 GHz FSR) spectrometer with sCMOS camera (Andor ZYLA-4.2P-USB3). The laser power measured at the sample was approximately 6 mW and the spectral resolution was evaluated as ∼0.9 GHz. Full spectrometer specifications are listed in Table 1.

The sCMOS output of the dual-stage VIPA spectrometer presents square patterns arising from different diffraction orders with Brillouin peaks along diagonals. Out of multiple diffraction orders, a single square with linear edges and uniform intensity was selected. Brillouin measurements of the gels were acquired with an exposure time of 3 s, as this was found to give the optimal trade-off between acquisition time and signal-to-noise ratio. All measurements were conducted at room temperature (20 °C), taken in triplicate at varying locations within the sample, and average frequency shifts were determined for each concentration. Brillouin peaks were identified and a Lorentzian fit was applied using a method previously developed in our lab [3].

### 2.3 Calibration

Spectral calibration was performed using known values of frequency shift for methanol and water (5.59 GHz and 7.46 GHz, respectively [9]). Calibration spectra from these standards were collected at the beginning and end of all experiments. The parameters from initial and final calibration spectra were averaged to account for drift during the experiment. To convert the peak position from a pixel to frequency scale, a scaling factor *P* was determined according to the relation:

(2) $$P=\frac{2\left(\nu_{w}-\nu_{m}\right)}{X_{m}-X_{w}},$$

where $\nu_{m}$ and $\nu_{w}$ are the known frequency shifts, and $X_{m}$ and $X_{w}$ the distance in pixels between peaks from neighbouring dispersion orders (Fig. 3), with subscripts *m* and *w* denoting methanol and water, respectively.

The free spectral range (FSR) was taken to be a summation of the measured distance between adjacent Brillouin peaks (X) and the frequency shift (ν) of the standards: FSR=2ν+PX (see Table 1).

The Brillouin frequency shift $\nu_{B}$ for each gelatin sample was hence determined from the FSR and the distance X between adjacent Brillouin peaks according to the expression:

(3) $$\nu_{B}=\frac{1}{2}\left(FSR-PX\right).$$

### 2.4 Longitudinal elastic modulus

The acoustic wave velocity V was determined from the Brillouin frequency shift based on the equation (valid for backscattering geometry):

(4) $$V=\frac{\lambda \nu_{B}}{2n},$$

where λ is the incident wavelength and n the refractive index of the sample. From these data, the longitudinal elastic modulus M' was derived according to the relation:

(5) $$M^{'}=\rho V^{2},$$

where *ρ* is the mass density of the sample. Refractive indices were measured using an Abbe refractometer, with distilled water as the calibration standard. Measurements revealed a linear relation described by n=0.00217x+1.331 (R^2^ = 0.996) where x is the solute concentration (w/w). Densities were calculated by assuming ideal mixing:

(6) $$\rho =\frac{\left(m_{w}+m_{s}\right)}{\left(\frac{m_{w}}{\rho_{w}}+\frac{m_{s}}{\rho_{s}}\right)},$$

where *m* is the mass. Density was taken to be 1.00 g/cm^3^ for water and 1.35 g/cm^3^ for dry gelatin [10]. The density-to-square refractive index ratio, which is relevant in the relation between longitudinal elastic modulus and the frequency shift (Eqs. (4), (5))), was found to vary between 0.5628 and 0.5582 g/cm^3^ hence accounting for only 0.8% change in the concentration range probed.
